# Aesthetic judgment of apparent motion: effects of movement smoothness, synchrony, and shape

**DOI:** 10.1007/s00426-025-02232-y

**Published:** 2026-02-24

**Authors:** Ernesto Monroy, Guido Orgs

**Affiliations:** 1 Department of Psychology, Universidad del Norte, Barranquilla, Colombia; 2 Department of Psychology, Brunel University of London, London, UK; 3 Institute of Cognitive Neuroscience, University College London, London, UK

**Keywords:** Empirical aesthetics, Method of choice, Body perception, Synchrony

## Abstract

Recently, Psychology of Aesthetics is starting to address the gap in knowledge on synchronous movement observation. This article presents two experiments that assess aesthetic judgments of animations. In Experiment 1, participants watched 32 video clips of human body animations depicting smooth synchrony, smooth asynchrony, abrupt synchrony, and abrupt asynchrony. Participants rated the videos using semantic differential scales of liking, arousal, variety, control, diversity, familiarity, obviousness, and happiness. In Experiment 2, participants watched 32 video clips of human and abstract animations depicting postural fluency, abstract fluency, postural disfluency, and abstract disfluency. Participants rated the videos using the same semantic differential scales of Experiment 1. In Experiment 1, our analysis revealed that synchrony enhanced the preference ratings for abrupt movements, while asynchrony diminished the preference ratings for smooth movements. In Experiment 2, participants’ responses showed that human body animations tend to be more calming when fluent and more exciting when disfluent, in comparison to abstract animations. In both experiments, participants liked movement smoothness and synchrony, and disliked abruptness and asynchrony. Findings from the two experiments highlight the aesthetic appeal of synchronous movement and the aesthetic strength of the human body in motion.

**Supplementary Information:**

The online version contains supplementary material available at 10.1007/s00426-025-02232-y.

Synchronous human movement is a common occurrence in both daily activities and in artistic performance, manifesting whenever a group of individuals engages in similar actions, such as commuters strolling in the same direction or dancers executing pirouettes simultaneously. Synchrony can vary in its degree, with some instances being less rigid, like pedestrians walking along a street, and others being more precise, as seen in highly choreographed performances. Regardless of the level of precision, synchrony is a fundamental and universal aesthetic characteristic present in collective human movement, spanning diverse cultural groups, ceremonies, religions, and dance forms (Christensen & Calvo-Merino, 2013). Thus, it is pertinent to explain the psychological basis of the appeal that emerges from such coordinated actions.

Synchronous movement has been explored within the field of social psychology, with a focus on the social advantages of engaging in or observing collective actions (Wiltermuth & Heath, 2009; Tarr et al., 2015; Eskenazi et al., 2015; Lakens & Stel, 2011). Recently, the psychology of aesthetics has begun to address the knowledge gap concerning the cognitive mechanisms underlying the aesthetics of observing actions that are performed in synchrony (Cracco et al., 2022; Monroy et al., 2022). Previous research has established that human body movement is a powerful medium for conveying emotional information. Seminal studies using point-light displays have demonstrated that observers can accurately recognize specific emotions solely from kinematic patterns, even in the absence of other visual cues (Dittrich et al., 1996). More recent work has expanded on this, providing validated stimulus sets of full-body dance movements to investigate how distinct emotions are perceived and categorized (Christensen et al., 2023).

This article presents two experiments that assess aesthetic judgments of human and abstract animations. We explored the effects of visual aesthetic features on participants’ aesthetic judgments. The visual features were movement synchrony/asynchrony, movement smoothness/abruptness, and human/abstract shapes. Participants’ aesthetic judgments were liking, arousal, variety, control, diversity, familiarity, obviousness, and happiness.

In the current experiments, we defined synchrony as the execution of actions with identical timing (Monroy et al., 2022). In this manner, the observed action will be more closely aligned with the deliberate synchrony found in military choreographies and artistic performances, including popular dance, folk dance, and classical ballet. Asynchrony was defined as the simultaneous execution of distinct movements (Monroy et al., 2022). This type of asynchrony is commonly observed in contemporary dance, among other styles. In other words, movement synchrony is conceptualized as the coordination of movements between individuals. Synchronous movements occur when dancers perform identical actions simultaneously, whereas asynchrony is exemplified by dancers executing distinct postures within the same frame.

While recent research has laid important groundwork, a critical gap remains in understanding how different perceptual features of group movement interact to shape aesthetic judgement. For instance, previous research has explored the aesthetic appeal of synchrony in feasible dance movements performed by humans (Monroy et al., 2022). In a related study, Cracco et al. (2022) used human body animations to show that the brain integrates form and motion cues, but their analysis was limited to aesthetic liking as a single measure. More recently, Cracco et al. (2023) compared the neural processing of human versus corkscrew animations, but this was focused on individual movement sequences and did not measure aesthetic responses.

Therefore, to our knowledge no study to date has systematically and simultaneously investigated the aesthetic impact of both group-level properties (synchrony vs. asynchrony) and individual movement features (smoothness vs. abruptness). Furthermore, it remains unknown how these judgments are modulated by the visual form of the agents (human vs. abstract) across a richer set of semantic ratings beyond liking. Our study was designed to explicitly address this multifaceted gap.

In the present two experiments, we measured participant’s aesthetic judgments when watching human and abstract animations. Our independent variables were movement continuation (smooth/abrupt), joint actions (synchrony/asynchrony) and shape (human body/abstract images). Here, smooth movement is a motion with good continuation. Motion smoothness involves a continuous, successive change between similar positions that progressively alter the posture. In contrast, an abrupt movement is characterized by poor continuity: a sudden, non-sequential shift between distinct postures, resulting in a rapid change in position and orientation. In this sense, smooth movements follow the gestalt principle of good continuation, meaning that a smooth movement is more predictable and easier to process than an abrupt motion. A detailed description of movement continuation can be found in Monroy et al. (2023).

Synchronous movement aligns with the gestalt principle of “common fate” (Arnheim, 1974; Koffka, 1935; Wertheimer, 1923/1938), which suggests that objects or individuals moving together in the same direction and at the same speed are perceived as a unified entity. Berlyne (1972) asserts that visual complexity increases with irregular arrangements and the diversity of elements (i.e., greater differences among elements in a configuration). Conversely, a regular arrangement with homogeneous elements results in redundancy (i.e., greater similarities between elements). Consequently, synchrony tends to be visually simpler to process than asynchrony, as synchronous movements are more redundant (identical movements/postures), whereas asynchronous movements are perceived as more complex (divergent movements/postures).

Studying the interaction between smooth/abrupt motion and synchrony/asynchrony is crucial, as certain aesthetic characteristics are more easily processed than others. Both smooth motion and synchrony are less demanding to process compared to abrupt motion and asynchrony. Based on processing fluency theory (Reber et al., 2004), we expect that participants would show a preference for watching movements that are both synchronous and smooth, as they are easier to process.

To test aesthetic judgments regarding images, our participants rated human body and abstract animations. Previous research has found a preference for human body animations (Monroy et al., 2023). However, that previous study applied the method of production, in which participants created the animations. In our two experiments, we applied the method of choice, in which researchers create the visual stimuli and participants observe the animations.

Building on prior research that connects motor familiarity with aesthetic preference (Kirsch et al., 2013, 2015), and in alignment with the embodied cognition framework (Glenberg & Kaschak, 2002; Glenberg et al., 2008), it is hypothesized that participants will favor scenes depicting human body actions over abstract videos. This, due to the ability to relate to the movements of others in relation to their own physical actions.

In the two experiments, participants watched video clips and reported their aesthetic judgments using semantic differential scales (Osgood et al., 1957; Berlyne, 1974). In this case, the observed features of the dance (synchrony/asynchrony, smoothness/abruptness) will influence the aesthetic judgment. Experiment 1 showed human body animations depicting joint actions. Experiment 2 showed human body animations and abstract animations performing joint actions.

To test our hypothesis, semantic differential scales were employed to assess participants’ aesthetic judgments regarding liking, arousal, variety, control, diversity, familiarity, obviousness and happiness. These semantic differentials were chosen since they have been utilized in prior studies for examining aesthetic judgment to artistic objects such as group dance (Monroy et al., 2022), paintings (e.g., liking, arousal, variety, control, obviousness, happiness; Tucker, 1955, as cited in Osgood et al., 1957), and dance movements (e.g., liking; Calvo-Merino et al., 2008). Smoothness and synchrony are likely to be perceived as associated to calmness, repetition, control, uniformity, familiarity, obviousness, and happiness. We hypothesize that participants will prefer processing information that can be characterized as redundant (Berlyne, 1972), such as repeated and uniform movements, which are typically linked to synchronous movements. Particularly in synchronized dances, performers are *repeating* the same movements, displaying a general *uniformity* in the movements. We anticipate that participants will favor movements that are obvious, familiar, and smoothly controlled. Obvious sequences are easily processed in comparison to subtle ones, familiarity enhances processing fluency (Reber et al., 2004), and controlled movements are more predictable and recognizable than accidental movements (Grossman & Blake, 2002; Hiris, 2007; Neri et al., 1998; Poom & Olsson, 2002; Pyles et al., 2007; Simion et al., 2008). In other words, the clarity, familiarity, and predictability of movements reduce perceptual uncertainty, thereby increasing fluency. Since positive valence is linked to processing fluency (Reber et al., 2004), we expect smooth synchrony to evoke calmness and happiness. Conversely, abruptness and asynchrony are anticipated to be less liked, being associated with qualities such as excitement, subtlety, unfamiliarity, diversity, sadness, and unpredictability, as these features are harder to process.

## Experiment 1

### Methods

#### Participants

Participants were first year psychology students from Brunel University London (*n* = 30, 29 female), age range (18–20, *M* = 18.90, *SD* = 0.80). Participants were non-experts in dance recruited through Brunel University participant pool system and received course credits for participation. The two experiments were approved by the Ethics Committee at Brunel University London. In Experiment 1, a total of 24 participants was calculated based on G*Power (Faul et al., 2007) for a medium effect size and a statistical power of 0.80.

#### Materials 

**Fig. 1:**
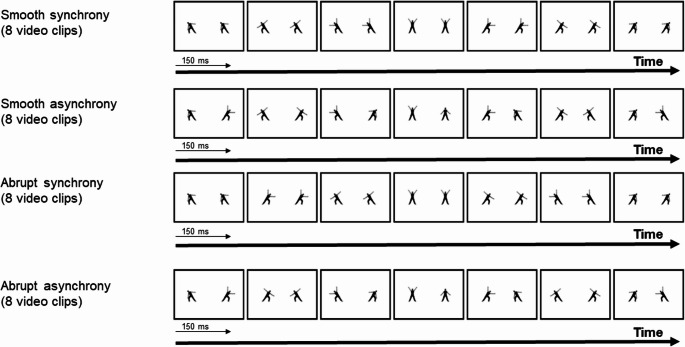
Example of frames used in Experiment 1. Frames depict human body animations with smooth synchrony, smooth asynchrony, abrupt synchrony, and abrupt asynchrony

In both experiments, the animations were projected on a 19-inch monitor, with a viewing distance of 50 cm. Each animation had a length of 18 cm and a width of 12 cm. In Experiment 1, the stimuli consisted of black-and-white muted video clips displaying four types of digital animations with two dancers (see Fig. 1). These video animations were based on similar human body back-view images used in previous studies (Orgs et al., 2013; Monroy et al., 2023). The body postures’ images were duplicated side-by-side, depicting two dancers at the same frame. A 2 × 2 factorial within-subjects design was employed to manipulate the interaction between synchrony (synchronous, asynchronous) and movement continuation (smooth, abrupt). The four resulting conditions were as follows:

(1) Smooth synchronous animations: Both performers adopted the same posture simultaneously, with a smooth transition between postures. (2) Smooth asynchronous animations: Each performer assumed different postures per frame and executed transitions with good continuation. (3) Abrupt synchronous animations: The performers maintained the same postures per frame and executed movements with bad continuation. (4) Abrupt asynchronous animations: The performers showed varying positions in each frame and executed movements with bad continuation.

The static scenes were edited using Microsoft Publisher and subsequently converted to animation format with Windows Movie Maker. Each one of the animations comprised a sequence of seven frames, repeated over six loops, with each frame shown for 150 milliseconds. A previous study has shown that participants like animated sequences when they are presented at this pace (Orgs et al., 2013). Thus, the total video duration was 6.3 s. In each condition, eight videos were shown (four oriented left to right and four in the opposite direction), resulting in a total of 32 videos for the experiment. The video stimuli used in Experiment 1 and Experiment 2 are available as Supplementary Materials.

Each animation was structured around a specific postural progression consisting of seven frames. The sequence followed a symmetrical spatial logic:

1. Initial Phase (Frames 1–3): Three postures where the body is standing but inclined toward one side (left or right), with variable arm movements in each frame.
2. Central Phase (Frame 4): A central standing posture with arms extended.
3. Final Phase (Frames 5–7): Three postures inclined toward the opposite side, again with variable arm movements.

Following a principle of seriation, the initial arm positions and subsequent postures were systematically varied for each animation. Half of the videos (4 per condition) started with an inclination to the left, and the other half (4 per condition) started to the right. It is important to note that manipulating movement continuation by varying the spatial displacement between frames while keeping temporal intervals constant necessarily alters the implied physical velocity of the movement. Abrupt changes involve larger displacements and are thus inherently “faster” in physical terms than smooth changes.

#### Measures

Eight 7-point semantic differential scales were utilized to assess participants’ aesthetic ratings for the dance video clips. Participants were instructed to use the scales to rate each clip according to the dance movements observed, disregarding the dancers’ clothing and the background. Eight items were listed below each video clip. Both video and semantic differential scale orders were randomized. The following judgments were evaluated: aesthetic liking (dislike – like), arousal (calming – exciting), variety (repeated – varied), control (accidental – controlled), diversity (uniform – diverse), familiarity (unfamiliar – familiar), obviousness (subtle – obvious) and happiness (sad – happy).

The aesthetic liking scale (dislike – like) was used to measure aesthetic preference for the video animations. Since previous studies (Berlyne, 1974; Christensen et al., 2014; Orgs et al., 2013) have identified valence and arousal as relevant judgments in aesthetic appreciation, happiness (sad – happy) and arousal (calming – exciting) scales measured these constructs. Variety (repeated – varied), diversity (uniform – diverse), control (accidental – controlled), and obviousness (subtle – obvious) scales were applied to assess participants’ aesthetic judgment of movement’s visual features. The familiarity scale (unfamiliar – familiar) tested whether participants were acquainted with the movements on display.

#### Background questionnaire

In light of previous studies suggesting that experts and novices differ in their perception of visual stimuli (Furnham & Walker, 2001; Hekkert & van Wieringen, 1996; Illes, 2008; Pihko et al., 2011; Uusitalo et al., 2009), a background questionnaire was administered to gather participants’ artistic background and demographic details, particularly focusing on dance experience and exposure. This questionnaire included demographic questions on age, gender, dance training, and familiarity with dance performances.

#### Procedure

The two experiments were laboratory based. For both experiments, stimuli were presented in an online platform (SurveyMonkey) to facilitate future online replications. Participants completed a printed informed consent form and received brief verbal instructions as well as an overview of the study’s purpose at the start of the experiment. Following this, 32 video animations were presented randomly, one at a time. Each animation was rated using the semantic differential scales that appeared beneath it. The order of videos and scales was randomized, and participants then filled out the background questionnaire. Afterward, participants were debriefed.

## Results

All 7-point scales were calculated with values ranging from − 3 to 3, where negative values represented proximity to one construct, while positive values indicated closeness to the opposite construct. To evaluate the impact of movement continuation (smooth/abrupt) and synchrony (synchronous/asynchronous) on aesthetic judgments, two-way repeated measures ANOVA were applied, as these conditions (smooth/abrupt; synchrony/asynchrony) were within-subjects. Consequently, two-way repeated measures ANOVA were chosen to analyze the effects. Statistically significant effects are summarized in Table 1. Non-significant effects are reported in Supplementary Materials.

**Table 1:** Summary of significant effects of continuation and synchrony on aesthetic judgments (Experiment 1)

Aesthetic Judgment	Effect	Wilks’ Lambda	F(df1, df2)	*p*	Partial eta squared
Liking	Continuation	0.55	23.48 (1,29)	< 0.001	0.45
	Synchrony	0.47	32.54 (1,29)	< 0.001	0.53
Arousal	Continuation	0.28	74.43 (1,29)	< 0.001	0.72
	Synchrony	0.66	14.99 (1,29)	< 0.001	0.34
	Interaction	0.75	9.92 (1,29)	< 0.05	0.26
Control	Continuation	0.40	43.39 (1,29)	< 0.001	0.60
	Synchrony	0.34	57 (1,29)	< 0.001	0.66
Variety	Continuation	0.58	20.94 (1,29)	< 0.001	0.42
	Synchrony	0.35	53.10 (1,29)	< 0.001	0.65
Diversity	Continuation	0.49	30.75 (1,29)	< 0.001	0.52
	Synchrony	0.41	42.36 (1,29)	< 0.001	0.59
Familiarity	Continuation	0.58	20.97 (1,29)	< 0.001	0.42
	Synchrony	0.46	33.60 (1,29)	< 0.001	0.54
Obviousness	Continuation	0.70	12.33 (1,29)	< 0.001	0.30
Happiness	Continuation	0.73	10.84 (1,29)	< 0.05	0.27
	Synchrony	0.80	7.36 (1,29)	< 0.05	0.20

Motion smoothness was liked over motion abruptness in conditions of synchrony and asynchrony. The effect of continuation on the mean liking judgment was significant, with participants showing a preference for smooth movements over abrupt ones, as previously noted. Similarly, the main effect of synchrony on mean liking judgment was also significant. Participants demonstrated a preference for synchrony over asynchrony.

**Fig. 2:**
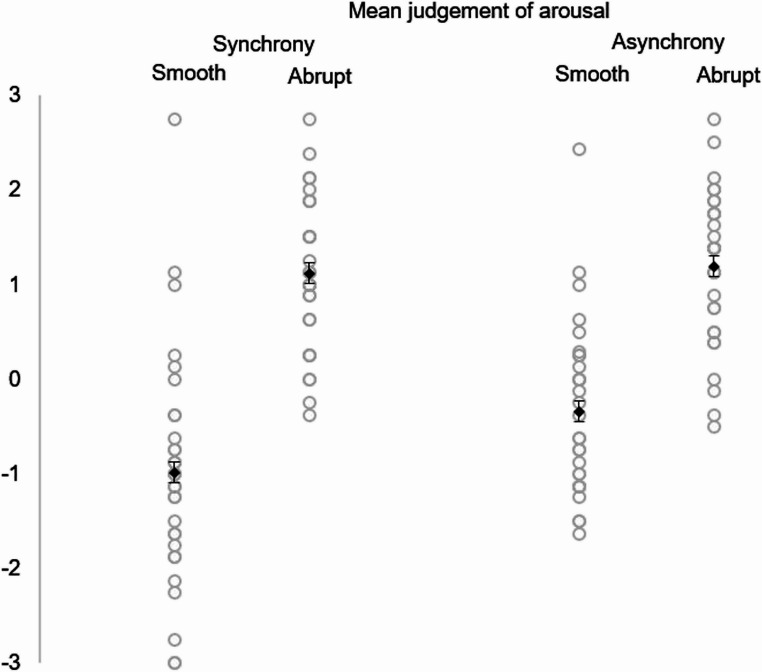
Judgment of arousal for movement continuation and synchrony (Experiment 1). Rhomboids represent mean scores. Error bars represent the standard error of the mean (SEM). Dots represent data points

The interaction of continuation and synchrony on judgment of arousal was statistically significant. Abruptness was judged as more arousing in conditions of synchrony and asynchrony. Nevertheless, asynchronous smoothness was deemed more arousing than those in synchrony (see Fig. 2). The effect of movement continuation on judgment of arousal was significant: abruptness was judged as more exciting than smoothness. Additionally, the main effect of synchrony on arousal judgment was significant. As indicated in the interaction effect, asynchrony was more arousing than synchrony, but this difference was significant only for motion smoothness. For abrupt movements, there was no significant difference in arousal between synchronous and asynchronous conditions.

In summary, abrupt movements, whether in synchrony or asynchrony, were similarly perceived as arousing. This indicates that for animations exhibiting abrupt movements, synchrony does not affect their excitement level, both will be judged as more exciting compared to smooth movements. However, for smooth movements, asynchrony will be more exciting than synchrony. This was confirmed by dependent t-tests, showing significant simple effects: smooth asynchrony (M = − 0.34, SD = 0.91) was significantly more exciting than smooth synchrony (M = − 0.98, SD = 1.24), t(29) = −3.92, *p* <.05, *r* =.59. There were no significant differences between abrupt asynchrony (M = 1.19, SD = 0.83) and abrupt synchrony (M = 1.12, SD = 0.81), t(29) = − 0.87, *p* =.40.

The continuation effect on control judgment was found to be significant. Movements that were smooth were judged to be more controlled compared to abrupt movements. Similarly, the synchrony effect on control judgment was also significant. Movements that were synchronous were regarded as more controlled compared to those that were asynchronous.

The continuation effect on variety judgment was significant. Abrupt movements were judged as exhibiting more variety than smooth movements. Likewise, the synchrony effect on variety judgment was also significant. Asynchronous movements were judged to exhibit greater variety than synchronous movements.

The continuation effect on diversity judgment was found to be significant. Abrupt movements were judged as having greater diversity compared to smooth movements. The synchrony effect on diversity judgment was significant. Asynchronous movements were evaluated as more diverse than synchronous ones.

A significant main effect of continuation on familiarity judgment was observed. Movements that were smooth were rated as more familiar compared to those that were abrupt. Similarly, the main effect of synchrony on the judgment of familiarity was also significant. Synchronous movements were judged as more familiar than asynchronous ones.

The effect of continuation on the judgment of obviousness was found to be significant. Abrupt movements were judged more obvious than smooth ones.

A significant main effect of continuation on the judgment of happiness was observed. Abrupt movements were rated as happier than smooth movements. Likewise, the main effect of synchrony on happiness judgment was significant. Movements in synchrony were judged as happier than movements in asynchrony.

## Interim discussion

Experiment 1 tested the effects of smooth/abrupt motion and synchrony/asynchrony on aesthetic judgments of human body animations. Our results support the hypothesis that non-experts tend to prefer watching smooth movements. As expected, judgments of variety, diversity, control and familiarity were associated to smoothness and synchrony, while the opposite (repeated, uniform, accidental, and unfamiliar) were attributed to abruptness and asynchrony.

Our results were in line with aesthetic theories posing that people prefer simple, familiar or easy to process stimuli, over complex, novel or disfluent stimuli. These theoretical positions include the mere exposure effect (Zajonc, 1968), Gestalt psychology (Arnheim, 1974), prototypicality (Martindale & Moore, 1988) and processing fluency theory (Reber et al., 2004). Aesthetic features like symmetry, repeated exposure, figure-ground contrast, and prototypicality contribute to fluency, ultimately leading to a preference for aesthetically pleasing objects. Viewing objects from a processing fluency standpoint, these frameworks suggest that individuals prefer aesthetic objects that are easy to process due to their simplicity, familiarity, and typicality. Our findings can be interpreted as simplicity being preferred over complexity, considering that synchronous and smooth movements can be described as less complex than asynchronous and abrupt movements (Berlyne, 1970, 1972).

In addition, previous research on static visual stimuli (Berlyne, 1974) and dance (Christensen et al., 2014) has associated high arousal to interestingness but not to liking. Our findings show that this is the case for aesthetic judgment of human body animations as well. Here, we found that the most exciting animations (abrupt movements) were different to the most liked (smooth synchrony).

Abrupt movements performed synchronously were judged as happier and more obvious than smooth movements performed synchronously. Specifically, synchronous movement was deemed happier than asynchronous motion. Interestingly, abruptness was rated as happier compared to smoothness. This could be explained by considering the arousal judgments. Abrupt movements (whether synchronous or asynchronous) were seen as the most exciting by respondents and were also judged as the happiest. This goes in line with a social psychology study that found an association between the meanings of happiness and excitement (Tsai et al., 2007). Our participants associated excitement (instead of calmness) to happiness. In other words, happy group movements are exciting actions, not calming performances.

Another unexpected result was that both synchrony and asynchrony were obvious to the respondents. Since participants watched two dancers, this could mean that observers could easily appreciate joint actions performed in duos. Future research could explore obviousness judgment with a higher number of dancers performing synchronous and asynchronous choreographies.

## Experiment 2

Experiment 2 tested if aesthetic perception of symmetry and fluency is specific to the shape of the human body, comparing abstract shape and human body animations. Smooth synchrony and abrupt asynchrony were chosen as conditions for this study, as these video animations elicited opposing trends in Experiment 1. Therefore, these two conditions are valuable for comparing the results of the Experiment 2 with those of Experiment 1.

Additionally, the comparison between abstract and human body animations is grounded in previous research linking motor familiarity to aesthetic preference (Kirsch et al., 2013, 2015) and aligns with the embodied cognition framework (Glenberg & Kaschak, 2002; Glenberg et al., 2008). Based on these studies, it is anticipated that participants will show a preference for human body animations over abstract animations, as they are likely to relate others’ body movements to their own. In Experiment 2, we used the same semantic differential scales from Experiment 1, as well as the same background questionnaire. According to this, we expected participants would like smooth synchrony and would dislike abrupt asynchrony. In addition, it was expected participants would prefer human body animations to abstract animations.

## Methods

### Participants

Participants were first year psychology students from Brunel University London (*n* = 19, 17 female), age range (18–20, *M* = 18.84, *SD* = 0.77). All participants were non-experts in dance recruited through Brunel University participant pool system and received course credits for participation. In Experiment 2, a sample size of 15 participants was calculated using G*Power (Faul et al., 2007), aiming for a large effect size and a statistical power of 0.95.

### Materials

**Fig. 3:**
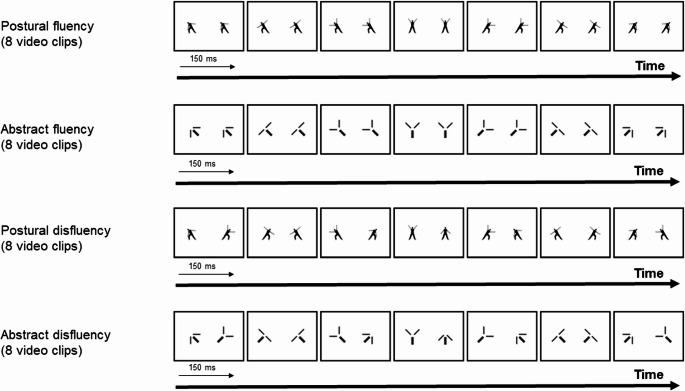
Example of frames used in Experiment 2. Frames depict animations with postural fluency, abstract fluency, postural disfluency, and abstract disfluency

Similar to Experiment 1, the stimuli in this experiment consisted of black and white video clips with muted colors, featuring four types of digital animations—two depicting abstract shapes and two showing dancers (see Fig. 3). The frames were edited and displayed in the same manner as in the previous experiment, maintaining consistent duration and orientation. These images were akin to those used in prior research (Orgs et al., 2013; Monroy et al., 2023). For Experiment 2, abstract animations were constructed to be structurally equivalent to the human stimuli, representing a simplified torso with two upper limbs that maintained the exact angles and proportions of the human figures. By generating these abstract stimuli from the identical motion sequences used for the human animations, we ensured that all core parameters, including trajectories, velocity, and temporal dynamics, were strictly matched. This design guarantees that every human sequence has a precise abstract counterpart with identical spatiotemporal properties, effectively isolating the influence of visual form from motion-related confounds.

For this experiment, two of the previous conditions (smooth synchrony and abrupt asynchrony) were displayed side by side, now featuring either two abstract shapes or two dancers. This setup created a 2 × 2 factorial within-subjects design, which examined the interaction between movement fluency (smooth synchrony, abrupt asynchrony) and image type (abstract animations, human body animations). Each video animation of the human body had a corresponding abstract animation that depicted the same sequence. The resulting four conditions were:

(1) Human body animations showing synchronous smooth movements. The two performers simultaneously assumed identical positions, with a smooth transition across positions (i.e., postural fluency). (2) Abstract animations showing synchronous smooth movements. The two abstract shapes simultaneously showed identical positions, with a smooth transition across positions (i.e., abstract fluency). (3) Human body animations showing asynchronous abrupt movements. The two performers adopted distinct positions in each scene, with abrupt transitions across positions (i.e., postural disfluency). (4) Abstract animations showing asynchronous abrupt movements. The two abstract shapes adopt distinct positions in each scene, with abrupt transitions across positions (i.e., abstract disfluency).

*Measures and Procedure.* The same scales, questionnaire, and procedure from Experiment 1 were employed in Experiment 2.

## Results

A two-way repeated measures ANOVA was conducted to examine the effect of movement fluency (fluent, disfluent) and shape (human body, abstract) on aesthetic perception. As in the previous experiment, these conditions (fluent/disfluent; body/abstract) were within-subject variables. Consequently, a two-way repeated measures ANOVA was chosen to assess this influence. Statistically significant effects are summarized in Table 2. Non-significant effects are reported in Supplementary Materials.

Fluent movements were preferred over disfluent movements in both postural and abstract animations. The main effect of movement fluency on the average liking judgment was significant, with participants showing a preference for fluent movements and a dislike for disfluent ones.

**Fig. 4:**
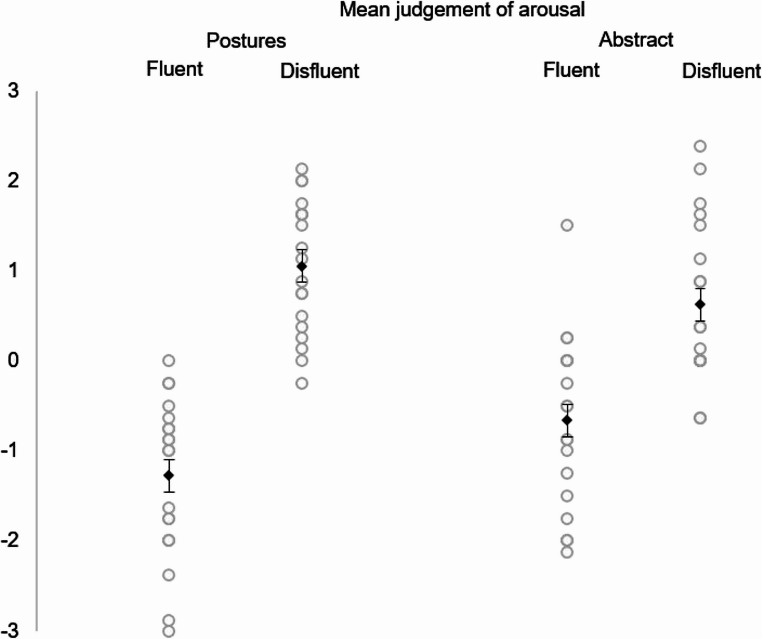
Judgment of arousal for movement fluency and shape (Experiment 2). Rhomboids represent mean scores. Error bars represent SEM. Dots represent data points

The interaction between movement fluency and shape in relation to the judgment of arousal was significant. Overall, disfluent movements were judged as more exciting for both human body and abstract animations. However, scores for human body animations were more extreme compared to abstract animations (see Fig. 4). Abstract fluency was found to be more exciting than postural fluency, while postural disfluency was perceived as more exciting than abstract disfluency.

The main effect of movement fluency on the judgment of arousal was also significant, with disfluent movements being judged as more exciting than fluent movements. Dependent t-tests confirmed significant simple effects. Postural disfluency (M = 1.05, SD = 0.74) was judged as more exciting than abstract disfluency (M = 0.63, SD = 0.90), t(18) = −2.74, *p* <.05, *r* =.54. Additionally, postural fluency (M = −1.28, SD = 0.89) was judged as more calming than abstract fluency (M = −0.66, SD = 0.96), t(18) = 3.59, *p* <.05, *r* =.65.

The main effect of movement fluency on the judgment of control was found to be significant. Movements that were fluent were judged as demonstrating greater control compared to disfluent movements. In addition, fluent animations were perceived as more familiar, whereas disfluent movements were judged as significantly more varied and diverse. No significant effects of fluency were observed for ratings of obviousness or happiness.

**Table 2:** Summary of significant effects of fluency and shape on aesthetic judgments (Experiment 2)

Aesthetic Judgment	Effect	Wilks’ Lambda	F(df1, df2)	*p*	Partial eta squared
Liking	Fluency	0.594	12.32 (1,18)	< 0.05	0.41
Arousal	Fluency	0.38	29.40 (1,18)	< 0.001	0.62
	Interaction	0.50	18.25 (1,18)	< 0.001	0.50
Control	Fluency	0.19	75.65 (1,18)	< 0.001	0.81
Variety	Fluency	0.23	61.25 (1,18)	< 0.001	0.77
Diversity	Fluency	0.32	38.16 (1,18)	< 0.001	0.68
Familiarity	Fluency	0.30	41.47 (1,18)	< 0.001	0.70

## Discussion

Experiment 2 tested how motion characteristics and stimulus shape affect aesthetic assessments in participants with no dance experience. The hypothesis that subjects would favor fluid movements was confirmed. Nevertheless, the expectation that human body videos should be liked over abstract animations was not supported, as no significant differences in preference ratings were observed between the two types.

Participants in Experiment 2 favored smooth movements over abrupt ones, although no notable differences were evidenced between abstract and body postures in terms of preference. Furthermore, the findings from Experiment 1 were replicated, with subjects showing a preference for smooth synchrony over abrupt asynchrony. Excluding evaluations related to obviousness and happiness, the aesthetic assessments supported the hypothesis differentiating fluent from disfluent movements.

The hypothesis concerning a distinction between abstract shapes and human body sequences was partially supported by a statistically significant interaction between movement fluency and shape, particularly in arousal ratings. Fluent body postures were perceived as more calming than abstract fluency, while disfluent body postures were deemed more arousing than disfluent abstract movements. This interaction underscores the aesthetic importance of human body movement: body animations were perceived as more calming when fluent and more exciting when disfluent, compared to abstract animations. Nonetheless, no significant differences were found on other aesthetic evaluation scales between abstract and body animations.

### General discussion

Experiment 1 explored the relationship between smooth motion and synchronous movement in human body video animations, while Experiment 2 investigated the interaction between action aesthetics (smooth/synchronous movement) and the visual form of the stimulus (abstract/body animations). Both experiments found that smoothness and synchronous movement significantly enhance aesthetic preferences, whereas abruptness and asynchrony notably reduce them. Other combinations resulted in intermediate effects. For example, in Experiment 1, smooth asynchronous movements and abrupt synchronous movements showed no significant difference in preference. As predicted, the smooth synchronous sequences were the most preferred, and the abrupt asynchronous sequences the least. However, smooth asynchronous and abrupt synchronous movements fell in the middle of the preference continuum. This unexpected finding, where the effect of synchrony was independent of smoothness, is consistent with hierarchical models of both dance (Orgs et al., 2013) music (Lerdahl & Jackendorff, 1983), suggesting that aesthetic effects at different hierarchical levels of temporal organization can operate independently. Smoothness relates to the local transitions between postures level, the transition between frames, while unison synchrony pertains to the simultaneity of these local transitions across multiple bodies. Our results indicate that novices assess human movement by considering all levels of movement sequences.

These findings are generally consistent with our hypotheses, though some were unexpected. For example, we anticipated synchronous and asynchronous smoothness to be preferred over synchronous and asynchronous abruptness. We had underrated the impact of synchronous movement combined with motion continuity. While we anticipated a difference between smooth asynchrony and abrupt synchrony, we found that synchrony increased the preference for abrupt movements and asynchrony decreased the preference for smooth movements, thus narrowing the gap between smooth and abrupt movements.

In Experiment 2, no significant differences were observed in most aesthetic judgments between abstract and body video animations. The only exception was in the assessment of arousal, where a significant interaction was found, with body video animations being more effective than abstract ones in conveying calmness and excitement through movement.

Our findings replicate and extend existing research using an apparent biological motion paradigm (Cracco et al., 2023). Our methodological approach, using apparent motion constructed from static frames, offers specific advantages over other common paradigms in biological motion research, such as point-light displays or full-motion video. This technique allows for precise, frame-by-frame manipulation of spatial and temporal parameters. Specifically, it enabled us to systematically construct and contrast conditions of smooth versus abrupt and synchronous versus asynchronous movement with a level of experimental control that is difficult to achieve with naturalistic recordings. This ensured that the effects of continuity and synchrony could be isolated without the potential confounds of individual stylistic variations found in continuous motion capture. However, we acknowledge that this experimental control comes at the cost of ecological validity. Unlike continuous motion capture data, our discrete apparent motion stimuli may reduce the richness of the kinematic information typically available in dance, potentially affecting the nuances of aesthetic appreciation.

Firstly, we found a preference for synchronous fluent movement over asynchronous disfluent movement. Moreover, we show that this preference for fluency is accompanied by perceived greater arousal for disfluent stimuli. Showed that disfluent movements were perceived as more complex than fluent ones. In our study, we show that they are also perceived as more exciting. Our study therefore links complexity of the movement sequence to the arousal of the viewer. We do not observe a significant effect of stimulus shape on preferences for apparent movements. While this seems surprising at first, it is a well-documented phenomenon. For instance, movement of abstract shapes and non-human agents can produce vivid illusions of animacy, and emotional expressions and intentionality can be perceived from the movements of abstract stimuli (Darda et al., 2024; Heider & Simmel, 1944; Press, 2011; Tidoni et al., 2024). Cracco et al. (2023) showed that animations of corkscrews can in fact produce larger evoked neural responses than those of the human body, but in contrast to ours, their study did not include any aesthetic ratings of these stimuli.

However, we found that stimulus shape did influence the effect movement fluency exerted on judgments of arousal, replicating earlier studies on body specificity of apparent movement that use either feasibility judgment (Shiffrar & Freyd, 1990, 1993; Orgs & Haggard, 2011) or indirect measures of time perception (Orgs et al., 2011, 2013, 2016). We note that the abrupt apparent movements performed by the human body are biomechanically impossible. As our introduction outlines, stimuli that are harder to process, because they are less clear, familiar, or predictable, tend to reduce processing fluency. The biomechanical impossibility of the abrupt human movements would place them in this category of low-fluency stimuli. Our data show that these same movements were judged as more exciting.

This finding is consistent with our initial hypothesis that features which are harder to process, such as abruptness, would be associated with higher arousal ratings like excitement. Thus, the low processing fluency inherent in these impossible movements provides a plausible account for why they were perceived as more exciting. In contrast, abrupt and fluent movement of abstract shapes are equally plausible because they are not constrained by the physical limitations of the human body and therefore do not produce significant difference in arousal judgments.

Our findings can also be interpreted through the lens of classic aesthetic principles, namely the “unity-in-variety” framework (Fechner, 1876) and the Gestalt law of Prägnanz (or “good figure”). The principle of Prägnanz posits that the perceptual system defaults to the simplest, most stable interpretation (for a review, see Van Geert & Wagemans, 2024). In our study, synchronous and smooth movements represent a more prägnant or “good” figure than asynchronous and abrupt ones, as they require less cognitive effort to process as a coherent event. Similarly, the “unity-in-variety” principle suggests that maximal aesthetic pleasure is found in stimuli that balance order with complexity. Our smooth synchronous condition maximizes “unity”, leading to high preference, while the abrupt asynchronous condition could be seen as maximizing “variety”, resulting in low preference.

Regarding the observed dissociation between happiness and liking, where abrupt movements were disliked yet rated as happier than smooth ones, our results align with previous findings in the field of emotion perception from biological motion. Previous research has demonstrated that kinematic features such as high velocity are primary cues for identifying happiness (Pollick et al., 2001). In our abrupt condition, the high apparent velocity inherently resulting from large spatial displacements likely triggered this semantic categorization of happiness. We propose that while high apparent speed drives the judgment of happiness, the lack of fluency (unpredictability and biomechanical impossibility) drives the negative aesthetic appraisal (dislike). This suggests a distinction between the cognitive recognition of an expressed emotion and the observer’s own hedonic experience, a dissociation that future studies could further explore by collecting phenomenological reports.

Future research could build on our findings in several key ways. First, it would be valuable to directly test the theoretical proposition that synchrony and smoothness are processed at different hierarchical levels, for instance by manipulating structural and dynamic features of movement independently. Second, establishing the generalizability of our results would require extending this research to more diverse participant populations, such as those varying in age or domain-specific expertise. Finally, complementing aesthetic ratings with additional dependent variables, such as measures of attention or memory for the stimuli, would offer a more comprehensive understanding of the cognitive and affective impact of visual synchrony and smoothness.

Furthermore, to build on our findings regarding expressiveness, future studies could incorporate a broader range of affect-related ratings beyond happiness and arousal. The collection of qualitative descriptions would also offer a richer, phenomenological layer to complement the quantitative data and deepen our understanding of the aesthetic experience. Additionally, future research should investigate the specific conditions under which these animations might be perceived as having life-like qualities versus being seen as purely mechanical. As noted in recent reviews (e.g., Parovel, 2023), kinematics play a powerful role in specifying animacy, potentially overriding form. Collecting qualitative reports would allow researchers to determine whether, for instance, abrupt human movements are perceived as “robotic” or if smooth abstract movements successfully evoke an impression of life.

In conclusion, the results of our two experiments reveal that novice participants prefer smooth and synchronous movements and judge human body and abstract animations in comparable, though not identical, manners. It was found that movement smoothness and synchrony significantly influenced aesthetic preference, supporting prior research on the preference for familiar and fluent movements (Kirsch et al., 2013, 2015; Monroy et al., 2022, 2023; Orgs et al., 2013; Topolinski, 2010). Additionally, we found that for novices, human body animations were more expressive than abstract animations in terms of aesthetic judgments of arousal, highlighting the unique expressive power of human body movement in non-verbal communication.

## Supplementary Information

Below is the link to the electronic supplementary material.

Supplementary Material 1 (XLSX 13.4 KB)

Supplementary Material 2 (XLSX 15.0 KB)

Supplementary Material 3 (DOCX 20.9 KB)

Supplementary Material 4 (ZIP 21.2 MB)

## Data Availability

The data collected and analyzed in the experiments is available as supplementary materials.
