# Supplementary material for: Aesthetic judgment of apparent motion: effects of movement smoothness, synchrony, and shape
Source: Psychol Res. 2026 Feb 24;90(2):40. doi: 10.1007/s00426-025-02232-y (PMC12932363; doi:10.1007/s00426-025-02232-y)
Supplement: Supplementary file 3 — Supplementary Material 3 (DOCX 20.9 KB) [file 426_2025_2232_MOESM3_ESM.docx]

**Non-significant effects (Experiment 1)**

- The interaction effect between continuation and synchrony regarding *judgement of liking* was not significant, Wilks’ Lambda = .93, *F* (1, 29) = 2.21, *p* = 0.15.
- The interaction effect between continuation and synchrony regarding *judgement of control* was not significant, Wilks’ Lambda = .92, *F* (1, 29) = 2.39, *p* = .14.
- The interaction effect between continuation and synchrony regarding *judgement of variety* was not significant, Wilks’ Lambda = .96, *F* (1, 29) = 1.19, *p* = .29.
- The interaction effect between continuation and synchrony regarding *judgement of diversity* was not significant, Wilks’ Lambda = .99, *F* (1, 29) = .32, *p* = .58.
- The interaction effect between continuation and synchrony regarding *judgement of familiarity* was not significant, Wilks’ Lambda = .98, *F* (1, 29) = .49, *p* = .50.
- The interaction effect between continuation and synchrony regarding *judgement of obviousness* was not significant, Wilks’ Lambda = .99, *F* (1, 29) = .25, *p* = .62. The main effect of synchrony on judgement of obviousness was not significant, Wilks’ Lambda = .99, *F* (1, 29) = .23, *p* = .63.
- The interaction effect between continuation and synchrony regarding *judgement of happiness* was not significant, Wilks’ Lambda = .99, *F* (1, 29) = .20, *p* = .66.

**Non-significant effects (Experiment 2)**

- The interaction effect between movement fluency and shape regarding *judgement of liking* was not significant, Wilks’ Lambda = .91, *F* (1, 18) = 1.79, *p* = .20.
- The main effect of shape on *judgement of arousal* was not significant, Wilks’ Lambda = .96, *F* (1, 18) = .71, *p* = .41.
- The interaction effect between movement fluency and shape regarding *judgement of control* was not significant, Wilks’ Lambda = .98, *F* (1, 18) = .38, *p* = .55. The main effect of shape on judgement of control was not significant, Wilks’ Lambda = .95, *F* (1, 18) = .92, *p* = .35.
- The interaction effect between movement fluency and shape regarding *judgement of variety* was not significant, Wilks’ Lambda = .99, *F* (1, 18) = .17, *p* = .68. The main effect of shape on judgement of variety was not significant, Wilks’ Lambda = 1.00, *F* (1, 18) = .01, *p* = .94.
- The interaction effect between movement fluency and shape regarding *judgement of diversity* was not significant, Wilks’ Lambda = .90, *F* (1, 18) = 2.00, *p* = .18. The main effect of shape on judgement of diversity was not significant, Wilks’ Lambda = .99, *F* (1, 18) = .13, *p* = .72.
- The interaction effect between movement fluency and shape regarding *judgement of familiarity* was not significant, Wilks’ Lambda = .92, *F* (1, 18) = 1.60, *p* = .22. The main effect of shape on judgement of familiarity was not significant, Wilks’ Lambda = 1.00, *F* (1, 18) = .05, *p* = .82.
- The interaction effect between movement fluency and shape regarding *judgement of obviousness* was not significant (Wilks’ Lambda = .91, *F* (1, 18) = .1.84, *p* = 19). The main effect of fluency on judgement of obviousness was not significant (Wilks’ Lambda = .85, *F* (1, 18) = 3.12, *p* = .09). The main effect of shape on judgement of obviousness was not significant (Wilks’ Lambda = 1.00, *F* (1, 18) = .06, *p* = .82).
- The interaction effect between movement fluency and shape regarding *judgement of happiness* was not significant (Wilks’ Lambda = .92, *F* (1, 18) = 1.53, *p* = 23). The main effect of fluency on judgement of happiness was not significant (Wilks’ Lambda = .83, *F* (1, 18) = 3.67, *p* = .07). The main effect of shape on judgement of happiness was not significant (Wilks’ Lambda = 1.00, *F* (1, 18) = .19, *p* = .67).
